# Palliative Treatment of Bowel Obstruction With Colostomy Under Local Anesthesia in Frail Patients: A Single-Site Experience

**DOI:** 10.7759/cureus.45698

**Published:** 2023-09-21

**Authors:** Christos Doudakmanis, Kyriaki Baxevanidou, Paraskevi Chatzikomnitsa, Christina Kolla, Konstantinos Bouliaris, Anargiros Giaglaras, Matthaios Efthimiou, Georgios D Koukoulis

**Affiliations:** 1 Department of Surgery, General Hospital of Larissa, Larissa, GRC; 2 Second Department of Propaedeutic Surgery, Laiko General Hospital of Athens, Athens, GRC

**Keywords:** critically ill patients, local anesthesia, colostomy, emergency surgery, malignant, large bowel obstruction

## Abstract

Introduction: Attendance of patients to the emergency department due to acute large bowel obstruction is a common phenomenon. Most of these patients are elderly, critically ill, and with high comorbidity. The literature suggests that more than 50% of these cases are due to colon cancer. Since this condition is considered to be an emergency, immediate intervention and response is imperative.

Purpose: The aim of the present study is to present our surgical technique of colostomy formation under local anesthesia in selected critically ill patients, with increased perioperative risk and acute large bowel obstruction.

Materials and methods: This is a retrospective study of 24 patients, with obstipation, who underwent emergency colostomy under local anesthesia, during the period from 2014 to 2021.

Results: The mean age of the patients was 77 years. The vast majority of patients had an American Society of Anesthesiologists (ASA) score of ≥3 and a Charlson score of ≥7. The most common colostomy was transverse colostomy (21/24 patients). The patients' hospitalization ranged from four to 42 days. Only one patient died. All colostomies functioned properly in the immediate postoperative period. Only one patient required postoperative admission to the ICU.

Conclusions: Colostomy under local anesthesia in critically ill, elderly patients is an alternative option for the treatment of ileus.

## Introduction

Acute large bowel obstruction is a surgical emergency that, on most occasions, requires immediate investigation and treatment. It is characterized by high morbidity and mortality, if not treated immediately. Bowel obstruction can be the result of either mechanical obstruction or extrinsic compression [1,2].

Conservative treatment of acute bowel obstruction is ineffective in most cases; therefore, a surgical approach should be considered. Initial conservative treatment may be helpful to prepare patients for the subsequent surgical procedure, in terms of hydration and correction of electrolyte disorders. The treatment of choice for patients with acute large bowel obstruction is surgery under general anesthesia. This type of surgery may include emergency, one- or two-stage colectomy (colectomy and anastomosis or Hartmann’s colectomy) [3]. While a resection is the best choice, it might not be a feasible approach, so a diverting colostomy could be the safest choice for the patient, in terms of postoperative quality of life [4].

Emergency surgery, in contrast to elective surgery, poses a greater risk for the patient, as the acute nature of the operation leads to minimum preoperative preparation. Elderly patients with many comorbidities are especially at risk of postoperative complications and morbidity [5].

A colostomy is preferably done under general anesthesia. In critically ill patients with acute bowel obstruction and multiple comorbidities, general or even epidural anesthesia could be inapplicable, especially in patients receiving anticoagulant therapy. This derives from the high mortality of endotracheal intubation, a rather stressful event, that could result in respiratory problems and sepsis. Mortality is extremely increased in critically ill patients, especially when the procedure is done under general anesthesia. This is attributed to the stress of general anesthesia in frail patients [6].

The formation of colostomy as proposed by Duret under local anesthesia is a technique widely used in pediatric surgery, as a salvage operation in neonates with intestinal malformation and colonic atresia. However, it is not well studied in adult populations. Recently, DiGiacomo et al. reported in 2021 that this procedure is feasible in selected patients and these colostomies tend to function normally [7].

The purpose of this study is to describe the surgical technique of colostomy formation under local anesthesia and present our results. Our primary endpoint is to present a feasible palliative surgical technique in selected frail patients. We must underline the fact that the purpose of this study is not to compare this method to the standard of care.

## Materials and methods

This retrospective observational study took place in 2021. For the purposes of our study, we analyzed the medical records of 679 patients, hospitalized in our surgical department between 2014 and 2021, due to bowel obstruction. We recorded all necessary data from medical records to analyze them further. The collected data included age, sex, American Society of Anesthesiologists (ASA) score, Charlson score, serum blood tests including electrolytes, renal and liver function tests, days of hospitalization, morbidity, and need for intensive care unit (ICU) admission. All patients were subject to preoperative assessment to ascertain that they were eligible to undergo surgery under general anesthesia and were categorized using the ASA and Charlson scores, thus stratifying their perioperative risk. The surgeon on-call, after providing adequate insight regarding a patient’s status and discussing therapeutic options with the patient and next of kin, proceeded to colostomy formation under local anesthesia in cooperation with the anesthesiologist on-call. A total of 24 patients were at an extremely high risk for endotracheal intubation and general anesthesia due to comorbidities and were subsequently treated with stoma under local anesthesia. We further analyzed collected data for these patients using descriptive statistics.

Surgical technique

Patients were positioned in the supine position. They were under constant monitoring from anesthesiologists, who administered intravenous analgesia, including paracetamol and opioids. Intravenous dexmedetomidine was also administered to four patients. All patients received supplemental oxygen and none of them required ventilatory assistance or intubation. All colostomies were performed under local anesthesia. Mean operative time was 25 minutes and there were no intra-operative complications.

Α solution of xylocaine (10 ml), ropivacaine (10 ml), and natural solution (10 ml) was administered intradermally, at the location of the incision. Α 3-4 cm incision was made on the abdominal wall. Additionally, the local anesthetic was administered subcutaneously and to the aponeurosis of the external oblique muscle. Next, an incision was made in the aponeurosis of the external oblique, the muscles were retracted, and the peritoneum was opened. A distended large bowel was subsequently recognized and using a Babcock clamp, the bowel was grasped, drawn to the abdominal wall, and then sutured to the aponeurosis of the external oblique, with 2-0 Vicryl sutures. Thereafter, 0 PDS (polydioxanone) sutures were used to diminish the size of the initial incision and reduce the risk of parastomal herniation occurrence. Finally, the antimesenteric aspect of the bowel was opened, bowel contents were removed using constant suction, and the bowel was fixed to the skin with 3-0 PDS sutures. Throughout the process, it is important to check the direction of the large bowel so that it does not twist.

## Results

Study population characteristics

In our surgical department, 24 patients were treated for bowel obstruction with colostomy under local anesthesia between 2014 and 2021. Twelve of the patients were male and 12 were female. All patients presented to the emergency department with neglected obstipation and bowel dilatation lasting at least three days. The most common presenting symptoms were multiple episodes of vomiting, dehydration, and oliguria due to acute renal impairment. After the first assessment of the patient’s personal and family history by treating physicians, complete laboratory tests were ordered, a nasogastric tube and urinary catheter were placed, and then a hydration treatment with Ringer’s lactate solution was initiated. Before the completion of the initial assessment, an electrocardiogram was done and cardiological and pulmonary consultations were requested when appropriate for stratification of the perioperative risk.

**Figure 1:**
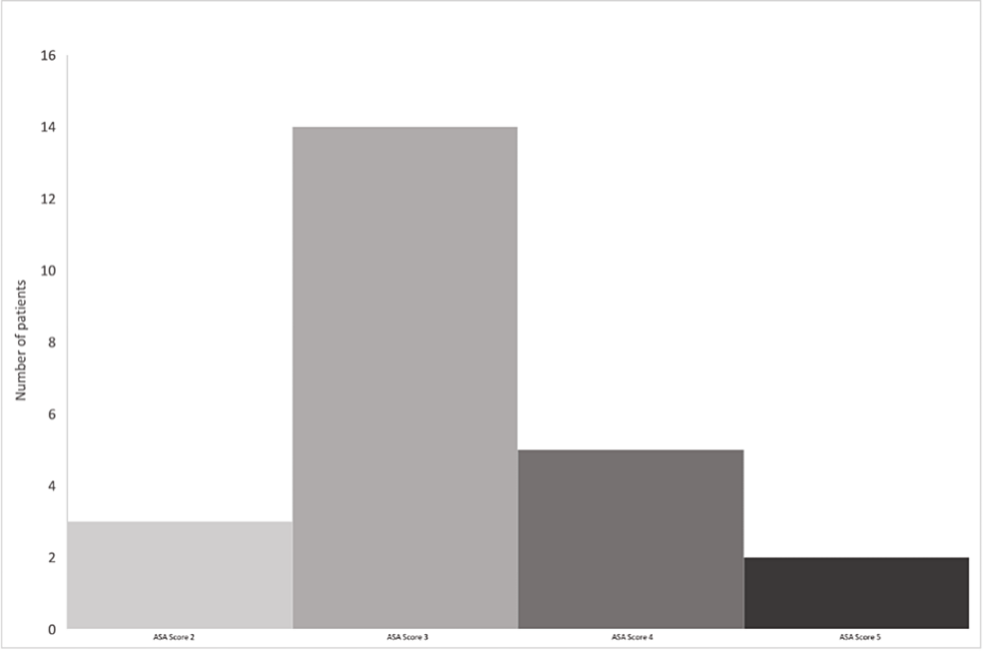
American Society of Anesthesiologists (ASA) score stratification of the 24 patients

Based on their ASA score, patients were stratified for the risk of complications following anesthesia (Figure 1).

Especially elderly patients with multiple comorbidities, such as renal function impairment, use of anticoagulants, or general debilitation, were considered as high risk for general or epidural anesthesia. Furthermore, proper hepatic function was affected in 13 out of 24 patients (54%), while 23 had an increased coagulation time (95%). All patients (100%) had leukocytosis at admission, while 19 (79%) of them had impaired renal function. Electrolyte disorders were found in 19 patients (79%). Characteristics of our patients are presented in Table 1.

**Table 1:** Characteristics of patients with bowel obstruction who were treated with colostomy under local anesthesia Data are expressed as mean ± sd and as n (%). ASA, American Society of Anesthesiologists; WBC, white blood cells; SGOT, serum glutamic oxaloacetic transaminase; SGPT, serum glutamic pyruvic transaminase; INR, international normalized ratio.

	Patients (n = 24)
Age, years ± sd	76.89 ± 12.59
Sex	
Male, n (%)	12 (50%)
Female, n (%)	12 (50%)
Stay, days ± sd	13.17 ± 9.48
Charlson score, mean ± sd	9.39 ± 3.36
ASA score, mean ± sd	3.17 ± 0.71
WBC, per microliter ± sd	17,629 ± 3,541
Sodium, mmol/L ± sd	145 ± 6
Potassium, mmol/L ± sd	2.84 ± 0.84
Urea, mg/dL ± sd	90.40 ± 17.36
Creatinine, mg/dL ± sd	1.78 ± 0.39
SGOT, IU/L ± sd	54 ± 24
SGPT, IU/L ± sd	33 ± 14
INR, mean ± sd	2.06 ± 0.30

Nineteen of our patients had locoregionally progressed neoplasms or distant metastasis at the time of diagnosis, while 10 of these patients were receiving chemotherapy, radiotherapy, or were under close follow-up by an oncologist.

**Figure 2:**
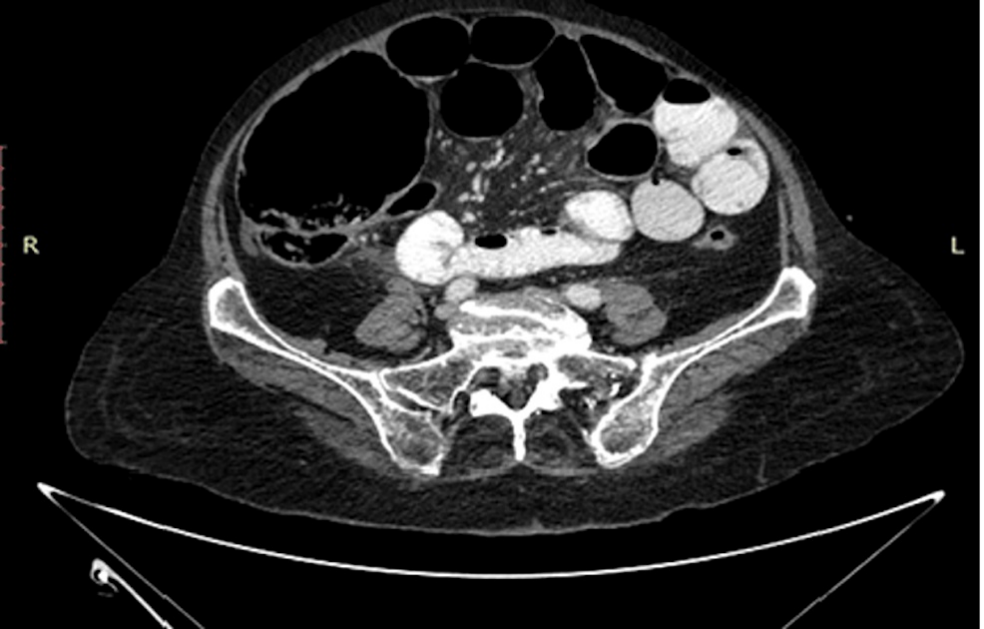
Representative CT image of the distended bowel as the result of obstruction

Upon admission, all patients were covered with broad-spectrum antibiotic therapy (cefoxitin 2 gr and metronidazole 500 mg). All patients underwent a computed tomography scan both for diagnostic reasons and for staging and preparation of the surgical approach. Figure 2 demonstrates a representative CT image.

Colorectal cancer was incriminated for the obstruction in 16 of the cases (67%). The cause of obstruction is shown in Table 2.

**Table 2:** Cause of obstruction Data are expressed as n (%).

	Patients (n = 24)
Colorectal cancer	
Transverse colon, n (%)	1 (4%)
Left colon, n (%)	5 (20%)
Sigmoid colon, n (%)	9 (38%)
Rectum, n (%)	1 (4%)
Uterine cancer, n (%)	4 (18%)
Renal cancer, n (%)	1 (4%)
Bladder cancer, n (%)	1 (4%)
Metastatic pancreatic cancer, n (%)	1 (4%)
Metastatic lung cancer, n (%)	1 (4%)

**Figure 3:**
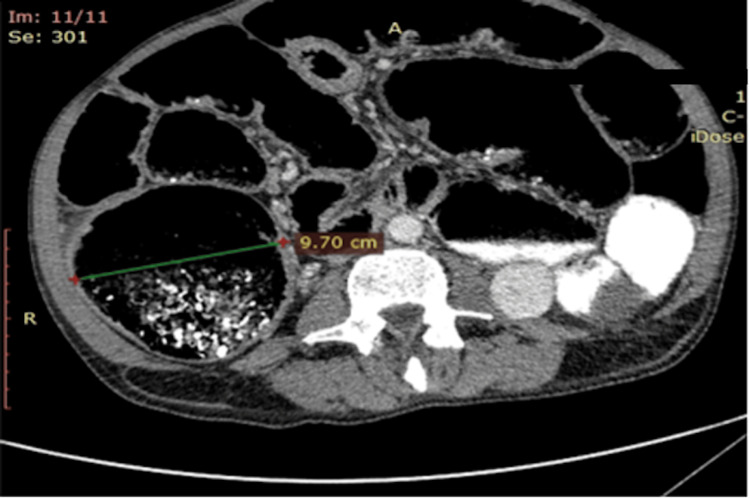
Distended cecum is shown in preoperative CT imaging in a case where cecostomy was preferred

Aside from colorectal cancer, obstruction was also the result of infiltration of neoplasms arising from nearby organs or due to distant metastasis from other organs. Conservative treatment was our first option, which in some cases lasted up to 48 hours, given that the patient's clinical status did not further deteriorate. During that period, patients were closely monitored. In most cases, emergency surgical intervention was required during the first 24 hours following admission. The indication for immediate surgical intervention was cecum distention of 10 centimeters or more, as calculated in computed tomography, to avoid the risk of cecum rupture (Figure 3).

Surgical procedure and postoperative course

The mean operative time was 25 minutes, and no significant intra-operative complications, like hemorrhage or infectious complications, were reported. The colon was identified with no difficulties and delivered to the skin with no tension. All colostomies functioned without complications within a few hours after surgery.

Twenty-one patients underwent colostomy of the transverse colon and in three patients, a cecostomy was preferred. The site of the colostomy was determined based on radiological findings, most importantly colon cancer location. In most cases, the right upper quadrant is preferred, as access to the proximate transverse colon is optimal in this area. The reasoning behind this is that there is less fat tissue so access to the peritoneal cavity is easier. In addition, the transverse colon usually lies right below the abdominal wall, without the interference of a distended small bowel, thus requiring minimum mobilization to perform the procedure.

Regarding postoperative complications, one patient presented dyspnea, tachypnea, and pleural effusion on the second postoperative day, hematochezia on the fourth postoperative day, and a blood transfusion was required. One patient developed a catheter-related bloodstream infection on the 16th postoperative day. Prolonged hospitalization was attributed to the impaired general condition of the patients, as more time was needed to correct electrolyte disorders and to recover from obstruction-derived malnutrition. No further early or late postoperative complications, stoma-related, were recorded.

The majority of patients were hospitalized in the surgical department following surgery, while only one patient required admission to the ICU. Out of the 24 patients, only one patient passed away in the ICU.

## Discussion

Advances in science and technology have offered surgeons better and more efficient techniques. Before local and general anesthesia and antibiotics, surgical minimalism was essential for the treatment of surgical emergencies.

In our study, most patients presented late to the emergency department in a critical state. Common symptoms included abdominal pain, constipation or obstipation, and abdominal distension [8]. A personalized approach was followed, based on each patient's status, disease extension, and radiological findings.

All selected patients were considered at high risk for complications when receiving general or epidural anesthesia, due to risk factors and comorbidities. Therefore, our team performed a colostomy under local anesthesia.

In the existing literature, there is little evidence regarding colostomy formation under local anesthesia, as only one study proposed this procedure as an alternative [7]. However, this procedure is well-studied in neonates and pediatric patients with colonic atresia and anorectal malformation, especially those considered high-risk for general anesthesia [9]. DiGiacomo et al. enrolled patients treated for sacral decubitus ulcers and these stomas were used to divert fecal matter from the ulcers. This study included data of only 11 patients, which underwent surgery at an elective setting to address non-malignant disease. This stands in contrast to our study, where we discuss the usefulness of a less invasive procedure to alleviate malignant bowel obstruction.

Notably, we performed stomas mainly in the right upper quadrant using the transverse colon, based on the reasons discussed above, when DiGiacomo et al. performed their colostomies in the left lower quadrant using the left and sigmoid colon. The use of the sigmoid colon for colostomy formation in the emergency setting may be challenging, as in most cases, it requires mobilization due to the dilation of the proximal colon, which is difficult to perform using only local anesthesia. Pellino et al. discussed the importance of decompression of the bowel in case of obstruction with minimal operating maneuvers. Their study proposed decompression through the appendix, with a procedure similar to appendectomy [10].

The current study is the outcome of a tremendous effort during times of uncertainty and pressure on healthcare systems worldwide. Our surgical team used a less invasive surgical technique to treat large bowel obstruction and, in many cases, advanced colon malignancies in an emergency setting. This is an easy-to-perform technique, with few steps and minimal differentiation required from patient to patient. We note that this is not a standard-of-care technique, but rather a life-saving procedure. During the pandemic, accessibility in healthcare systems was low, and delayed access had a negative impact on patients’ outcomes [11]. This comes in accordance with our data, as our patients had electrolyte disorders, acute renal failure, and coagulation deficiencies.

In this challenging period, when the availability of intensive care units reached an all-time low and with limited resources, the proposed operation helped our team treat patients with severe morbidity and otherwise high mortality, avoiding ICU admission [12]. This being said, only one of our patients required admission to the ICU. Therefore, our study suggests the use of colostomy under local anesthesia as an alternative treatment for large bowel obstruction, with acceptable morbidity and mortality, on selected critically ill, elderly patients. This technique ensures immediate decongestion of the bowel, treating the life-threatening condition of the patient. It could also be used as proper preparation of the patient for the final surgery, when feasible.

Our study comes with some limitations. Firstly, it is a retrospective study, so our analysis is based on available data from medical records. Only 24 patients were enrolled in a single group, and while the number of patients was sufficient to provide us with some insight into the usefulness of our method, it did not allow us to perform analysis to compare the method to other treatment options. Further future investigation with a randomized trial is considered necessary to draw conclusions.

## Conclusions

Colostomy under local anesthesia is a highly efficient and safe technique for critically ill patients, who present a high risk for general anesthesia, due to their comorbidities and their general extreme condition. This technique alleviates symptoms in the acute setting, by acting as a damage-control operation, providing the time needed to address patients’ comorbidities or even prepare them for definite surgical treatment. It must be noted that by avoiding ICU admissions, patients avoid prolonged hospitalization, which in most cases would be fatal due to the severity of their general condition.
